# A Single-Center Retrospective Study on Early Treatment for COVID-19 in Solid Organ Transplant Recipients During the Omicron Era: Outcomes and SARS-CoV-2 Viral Kinetics

**DOI:** 10.3390/microorganisms13081872

**Published:** 2025-08-11

**Authors:** Eugenia Milozzi, Elisa Biliotti, Alessandro Caioli, Valentina Mazzotta, Laura Loiacono, Silvia Meschi, Alessia Rianda, Andrea Antinori, Fabrizio Maggi, Gianpiero D’Offizi

**Affiliations:** 1 Infectious Diseases and Hepatology Unit, National Institute for Infectious Diseases Lazzaro Spallanzani—IRCCS, 00149 Rome, Italy; 2 Department of Epidemiology, National Institute for Infectious Diseases Lazzaro Spallanzani—IRCCS, 00149 Rome, Italy; 3 Regional AIDS Reference Centre, National Institute for Infectious Diseases Lazzaro Spallanzani—IRCCS, 00149 Rome, Italy; 4 Laboratory of Virology, National Institute for Infectious Diseases Lazzaro Spallanzani—IRCCS, 00149 Rome, Italy; 5 Health Direction Unit, National Institute for Infectious Diseases Lazzaro Spallanzani—IRCCS, 00149 Rome, Italy

**Keywords:** COVID-19, vaccination, SARS-CoV-2 viral kinetics, solid organ transplant recipients, monoclonal antibodies, antiviral agents, early therapy

## Abstract

Solid organ transplant recipients (SOTRs) are at high risk of severe coronavirus disease 2019 (COVID-19), therefore early treatment of mild infections is crucial to prevent increased morbidity and mortality. The effectiveness of early treatment in SOTRs has yet to be fully characterized due to the emergence of new SARS-CoV-2 variants and to COVID-19 vaccination implementation. The aim of this single-center retrospective study is to evaluate the outcomes, safety and impact on SARS-CoV-2 viral load kinetics of COVID-19 early treatment in SOTRs. The study includes 80 SOTRs with a laboratory-confirmed diagnosis of symptomatic SARS-CoV-2 infection enrolled between January and October 2022 and treated with either monoclonal antibodies or antivirals. All patients received COVID-19 vaccination and 68.8% of them showed detectable levels of anti-spike (S) antibodies. The occurrence of clinical events (hospitalization, intensive care unit admission, or death) was assessed within 30 days after treatment initiation. The quantification of SARS-CoV-2 viral load were performed at baseline and at day-7. The rate of hospitalization was 2.5% [0.3–9%] and no deaths occurred. All patients completed treatment with no serious adverse events. Median viral load decrease was 0.48 [0.26–0.69] log_2_ cycle threshold (ct) values, with no significant differences between SOTRs treated with monoclonal antibodies and those treated with antivirals. Viral load decrease was significantly associated with positive anti-s serology at baseline (β = 0.196, *p* = 0.01), number of days between symptom onset and treatment (β = 0.05, *p* = 0.03) and the number of comorbidities (β = −0.05, *p* = 0.03). We provide evidence of real-world effectiveness of early therapy in SOTRs infected with SARS-CoV-2 and demonstrate the relevant role of humoral response to vaccination in enhancing early viral load decay during treatment.

## 1. Introduction

Solid organ transplant recipients (SOTRs) have increased morbidity and mortality due to coronavirus disease 2019 (COVID-19) compared to the general population. Hospitalization rates of 50–70%, intensive care unit (ICU) admission rates of 20–30% and mortality rates of 10–30% have been reported in previously published studies [1,2,3]. The severe outcomes of COVID-19 disease in SOTRs are mainly due to chronic immunosuppressive therapy that prevents organ rejection, the presence of multiple comorbidities, older age, and reduced response to COVID-19 vaccination [4].

As the COVID-19 pandemic evolved, the availability of vaccination, the spread of the Omicron variant, and the introduction of early treatments for mild to moderate COVID-19 allowed better disease management, leading to a dramatic reduction in hospitalization and mortality rates in SOTRs [5,6,7]. However, SOTRs continue to be at higher risk of developing breakthrough severe acute respiratory syndrome coronavirus-2 (SARS-CoV-2) infections and poor outcomes of COVID-19 compared to healthy individuals [8,9,10].

Early outpatient treatment for mild to moderate COVID-19 includes anti-spike (S) monoclonal antibodies (mAbs) and direct antivirals. Real-world studies and case series show that early treatments are safe and effective in preventing the progression of SARS-CoV-2 infection to severe COVID-19 disease in SOTRs [6,11,12]. In the Omicron era, characterized by the rapid and ongoing emergence of new variants, many aspects of the effectiveness of COVID-19 early therapies in SOTR need to be highlighted. Furthermore, the assessment of the early kinetics of SARS-CoV-2 viral load in naso-pharingeal swabs (NPS) in vaccinated SOTRs who receive early treatment for COVID-19 may contribute to characterize drug effectiveness, given the low rate of severe clinical events in this scenario [6,13].

The study aims to evaluate the clinical outcomes (hospitalization, intensive care unit admission and death), the SARS-CoV-2 early viral load kinetics and their associated factors following early treatment for mild to moderate COVID-19 in SOTRs during the Omicron surge.

## 2. Materials and Methods

We conducted an observational single-center, retrospective study on all consecutive SOTRs with a laboratory-confirmed diagnosis of symptomatic SARS-CoV-2 infection enrolled within the early treatment access program of Lazzaro Spallanzani Institute between January and October 2022.

All included individuals have signed a written informed consent to participate in the study. The observational study protocol and the informed consent have been approved by the Ethical Committee of the National Institute for Infectious Diseases Lazzaro Spallanzani (Approval Number: n. 380, 09/30/2021. FAV del Registro delle Sperimentazioni 2020/2021).

Patients were treated with mAbs (bamlanivimab/etesevimab, casirivimab/imdevimab, sotrovimab and tixagevimab/cilgavimab) or antivirals (molnupiravir, remdesivir and nirmatrelvir/ritonavir) according to the AIFA (Agenzia Italiana del Farmaco) eligibility criteria (AIFA 2022), the physician’s evaluation, the availability of drugs over time and the presence of contraindications to specific drugs.

Outpatient visits were performed at baseline (day of treatment, day 1) and after 7 days (day 7), and consisted of a medical evaluation, vital signs recording and laboratory tests. Data on vaccination were extracted from the regional register (Anagrafe Vaccinale Regione Lazio). The occurrence of clinical events within 30 days after starting treatment was assessed by means of a telephone visit at day 30.

SARS-CoV-2 viral loads in NPS samples were quantified using the Abbott Alinity mReal Time System (Abbott Laboratories) on day 1 and day 7 and expressed as log2 of cycle threshold (Ct) values [14]. The use of Ct values as surrogate for viral load is consistent with other published studies [13,15,16], and its clinical significance has been discussed by Finks et al. [17]. Log2 transformation was adopted since the distribution of the viral load in the raw scale was positively skewed and significantly deviating from the normal distribution. This transformation helped to stabilize variance and improve the distributional properties of the data, facilitating parametric analysis, and it is consistent with the methodology used in a previously published study [13]. Identification of SARS-CoV-2 variants was performed by Sanger sequencing of the Spike coding gene on samples collected on day 1 using the ABI 3500 analyzer (Applied Biosystem) [18]. SARS-CoV-2 serology was performed by two chemiluminescence microparticle assays (CMIAs) detecting anti-Nucleoprotein (N) and anti-S/Receptor-Binding Domain (RBD) Immunoglobulins G (IgG) (ARCHITECT SARS-CoV-2 IgG and ARCHITECT SARS-CoV-2IgG II Quantitative, Abbott Laboratories, respectively). According to the manufacturer’s instructions, for the two CMIAs, Index > 1.4 and Binding Antibody Units (BAU)/mL ≥ 7.1 were considered positive for anti-N and anti-S/RBD IgG, respectively, as reported in previously published studies [13,19].

The primary endpoint was the proportion of SOTRs who experienced COVID-19-related clinical failure, defined as hospitalization, ICU admission or death due to COVID-19 within 30 days from early treatment administration.

The secondary endpoint was the assessment of SARS-CoV-2 viral loads variation between day 1 and day 7 and the identification of variables significantly associated with this variation.

Data were displayed as the median (interquartile range) for continuous variables and as an absolute number (percentage) for categorical variables. Differences in continuous variables were tested for significance using the *t*-test or the Mann–Whitney U test, depending on the distribution of the variable (evaluated via Q-Q plots and the Kolmogorov–Smirnov test); for categorical variables, the Chi Square test of independence or Fisher’s exact test were used. Analysis of Variance (ANOVA) was used to assess the significance of the differences between more than two groups. Effect sizes, calculated using Cohen’s d, have been reported for all statistically significant differences and have been interpreted according to established thresholds [20].

Simple linear regression models were run on the viral load variation between day 7 and day 1. Variables resulting significantly associated with viral decay in the univariable regression models were included in a multiple regression model adjusted for sex, age and type of treatment (monoclonal antibodies vs. antiviral drugs). To assess the robustness of the multiple regression model and to account for reported discrepancies between the two methodologies (i.e., Lord’s paradox) [21], a sensitivity analysis was performed where the same variables were tested for significance in an ANCOVA model with day 7 viral load as dependent variable and day 1 viral load as covariate.

R software version 4.1.2 [22] was used to perform the statistical analysis and to create all figures and tables.

## 3. Results

80 SOTRs were included in the study. The sample characteristics are summarized in Table 1.

The median age was 57 [48–65.5] years, 60% (N = 48) were males and the median BMI was 24.5 [21.8–27.3] Kg/m^2^. The median number of comorbidities was 1 [0–2], with the most frequent being chronic kidney disease, followed by diabetes and cardiovascular diseases. The median time from transplant was 5 years [3–10.5]; only 10% (N = 8) of the patients underwent transplantation <= 12 months before the day 1 visit. The type of transplant was kidney in 62.5% (N = 50) of the patients, liver in 25% (N = 20), heart in 8.8% (N = 7), liver/kidney in 2.5% (N = 2), and kidney/heart in 1.3% (N = 1).

Immunosuppressive treatment consisted of a single drug in 22.5% (N = 18) of the patients, a combination of two drugs in 35% (N = 28) of the patients and a combination of three drugs in 38.8% (N = 31) of the patients. Three patients (3.7%) were not undergoing immunosuppressive treatment during the study period. Calcineurin inhibitors were included in the immunosuppressive regimen in 82.5% (N = 66) of the patients, mycophenolate mofetil (MMF) in 57.5% (N = 46), steroids in 56.3% (N = 45) and kinase inhibitors in 12.5% (N = 10). At baseline, median estimated glomerular filtration rate (e-GFR) was 52.0 mL/min/1.73 m^2^ [38.6–71.1], median aspartate aminotransferase (AST) and alanina aminotransferase (ALT) levels were 26 [20–34] U/L and 19 [12.2–35] U/L, respectively.

All patients had received at least two doses of COVID-19 mRNA vaccination. Specifically, 8.8% (N = 7) received two doses, 62.5% (N = 50) received three doses and 28.8% (N = 23) received four doses (Table 1). The median time since the last dose of vaccine was 122 [90.5–156.5] days. Fifty-five patients (68.7%) (N = 25) had detectable levels of anti-S antibodies. Four patients (5%) tested positive for anti-N antibodies, while for 4 patients (5%) this information was missing.

SARS-CoV-2 variants were identified for 43 patients (53.7%), the majority (N = 25) consisting of B.1.1.529 followed by BA.4/BA.5 (N = 10), BA.2 (N = 6) and B.1.617 (N = 2).

In total, 62 patients (77.5%) received mAbs and 18 (22.5%) antiviral drugs (Table 2). Specifically, early SARS-CoV-2 treatment consisted of Sotrovimab in 65% of the cases (N = 52), Molnupiravir in 16.3% (N = 13), Bamlanivimab/Etesemivab in 6.3% (N = 5), Remdesivir in 5% (N = 4), Tixagevimab/Cilgavimib in 3.8% (N = 3), Casirivimab/Imdevimab in 2.5% (N = 2) and Nirmaltrelvir/Ritonavir in 1.3% (N = 1). The median time from onset of symptoms to treatment was 3 [2–4] days. All patients completed treatment.

**Figure 1:**
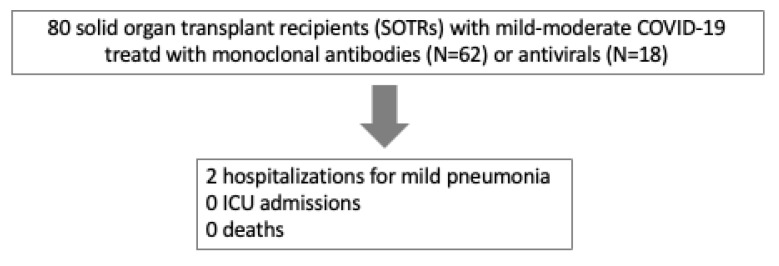
Patient flowchart showing clinical outcomes.

The rate of hospitalization within 30 days of starting early treatment was 2.5% [0.3–9%] (N = 2/80): 1 patient in the Sotrovimab group and 1 patient in the Molnupiravir group. In both these cases, interstitial pneumoniae was present but no oxygen therapy was needed and both patients were discharged from the hospital without relevant sequelae (Figure 1).

The first patient received 4 doses of COVID-19 mRNA vaccination against SARS-CoV-2 and anti-S antibodies were positive; the second patient received 3 doses of mRNA vaccination but tested negative for anti-S antibodies.

No ICU admission or death for all causes occurred during the 30 days following the infection.

The treatment was safe, since no patients experienced serious adverse events. Moreover, no significant changes in median ALT levels (19 vs. 17 U/L, *p* = 0.3) and e-GFR values (52.0 vs. 57.3 mL/min/1.73 m^2^, *p* = 0.67) were recorded from baseline to day 7.

All SOTRs obtained a negative antigenic NPS result; the median time from treatment to a negative swab result was 13 [9.7–16.2] days. The baseline median viral load was 3.98 [3.85–4.27] log2 Ct. The median difference between VL at baseline and VL at day 7 was 0.48 [0.26–0.69] log2 Ct (*p* < 0.001, d = 1.5). At baseline, patients treated with antiviral drugs showed comparable levels of viral load to those treated with mAbs (3.9 vs. 4.0 log2 Ct, *p* = 0.22). After 7 days, no significant differences were observed between the two treatment groups both in terms of time to negative swab (14 vs. 13 days, *p* = 0.32) and viral loads decrease (0.46 vs. 0.48 log2 Ct, *p* = 0.96).

Simple linear regression analysis on variables associated with the viral load change score (day 7–day 1) was performed and demonstrated that positive anti-S serology (β = 0.196, *p* = 0.01) and the number of days between symptoms onset and day 1 visit (β = 0.05, *p* = 0.03) were significantly associated with increased viral decay after treatment, while the number of comorbidities was associated with reduced viral decay (β = −0.05, *p* = 0.03). In multiple linear regression, after adjusting for sex, age and type of early treatment (mAbs vs. antiviral drugs), all three variables remained significant (Table 3).

A sensitivity analysis to confirm these results was performed using ANCOVA; in this case, the viral load at day 7 was used as a dependent variable, and day 1 viral load was included as a covariate (Table S1). The results of the ANCOVA confirmed the results obtained with the linear regression model run on the VL change score.

**Figure 2:**
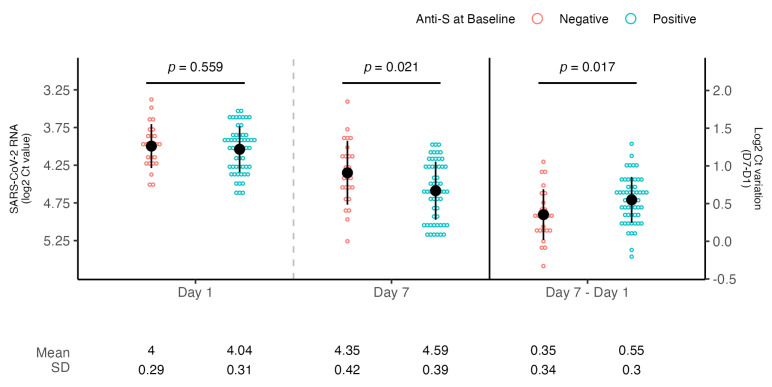
**SARS-CoV-2 RNA levels according to anti-S serology at baseline.** Dot plots showing the comparison of viral loads detected at day 1 and day 7 and mean SARS-CoV-2 RNA decrease according to baseline anti-S serology (negative or positive). Viral RNA levels are expressed as log2 Ct values. The mean of log2 CT values and SD are shown. Student’s *t*-test was used for comparison.

Patients with negative anti-S serology at baseline showed comparable levels of mean baseline viral load when compared to patients with positive serology (4 vs. 4.04 log2 Ct, *p* = 0.56) but a significantly reduced viral load decrease (0.35 vs. 0.55 log2 Ct, *p* = 0.02, d = 0.62) (Figure 2).

**Figure 3:**
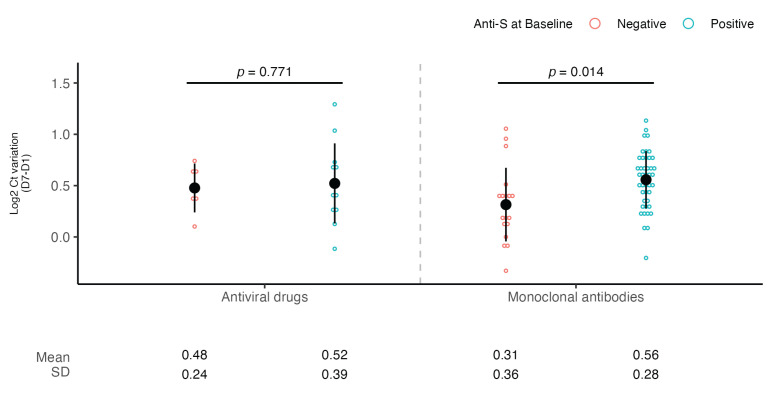
**SARS-CoV-2 viral load decrease according to anti-S serology at baseline and type of treatment.** Dot plots showing the comparison of viral load decrease between day 1 and day 7 according to type of treatment (antivirals or mAbs) and baseline anti-S serology (negative or positive). Viral RNA levels are expressed as log2 Ct values. The mean of log2 Ct values and SD are shown. Student’s *t*-test was used for comparison.

A subsequent subanalysis showed that a subject with positive anti-S serology experienced a significantly higher viral load decrease compared to a subject with negative anti-S serology when treated with mAbs (0.56 vs. 0.31 log2 Ct, *p* = 0.01, d = 0.79) but not with antivirals (0.52 vs. 0.48 log2 Ct, *p* = 0.77) (Figure 3).

## 4. Discussion

In this single-center retrospective study, we demonstrate that COVID-19 vaccination and early treatment are effective and safe in preventing hospitalization and mortality in SOTRs with mild to moderate COVID-19. We also report that early viral load decrease is enhanced by baseline anti-S antibody positivity, a marker of response to COVID-19 vaccination. Finally, SOTRs with positive anti-S serology experienced higher early viral load decrease compared to those with negative anti-S serology when treated with mAbs but not with antivirals.

Vaccine-induced SARS-CoV-2 humoral immune responses are attenuated in SOTRs due to the immunosuppressive medications taken to prevent organ rejection. However, several studies demonstrated that SARS-CoV-2 vaccination reduces the severity, hospitalization rate and mortality in SOTRs with Delta and Omicron breakthrough infections [23,24,25] and vaccine booster doses further reduce the rate of severe COVID-19-related outcomes in SOTRs [26].

In our study, all SOTRs were vaccinated, and most of them (91.2%) received three or four doses of a m-RNA vaccine with a median time since the last dose of vaccine of 4 months. Although the present study was not designed to evaluate the seroconversion rate after COVID vaccination in SOTRs, most of our subjects (68.8%) had detectable levels of anti-S antibodies at baseline. This finding is similar to that reported in the literature, since the seroconversion rate after three or four doses of SARS-CoV-2 vaccination ranges between 55 and 100% after 4–6 months [27,28]. Notably, liver transplant recipients show a higher immune response compared to other SOTRs [29], while older subjects and those on MMF treatment have a lower seroconversion rate [19,30,31].

Given the suboptimal immune response to COVID-19 vaccination, SOTRs continue to be a group at higher risk of breakthrough SARS-CoV-2 infection and poor COVID-19 outcomes compared to healthy individuals [8,9]. Early treatment of mild to moderate COVID-19 disease with antivirals or mAbs results in a reduction of morbidity and mortality in SOTRs [6,11,12,25]. In our study, the majority of SOTRs (77.5%) received mAbs and sotrovimab was administered in almost all cases (83.4%),within a median time of 3 days from the onset of symptoms. Although there are no randomized controlled trials (RCTs) that specifically evaluate the efficacy and safety of mAbs in SOTRs, the published observational studies revealed that SOTRs who received an early treatment with mAbs experience rates of hospitalization of 0–16.7%, ICU admission of 0–8.3% and mortality of 0–4.2% which are significantly lower than previous historical cohorts or SOTRs who did not receive early treatments for SARS-CoV-2 infection [32]. In the present study, hospitalization rate was 2.5% since two SOTRs were hospitalized for mild pneumonia, no patient required ICU admission, and no deaths occurred during the 30 days following early treatment administration. The observed encouraging clinical outcomes may be due to the high proportion of enrolled patients who have received ≥ 3 doses of vaccine and to the timely administration of early treatment (median time of 3 days from the onset of symptoms), which could play an important role in the setting of transplantation. Direct antivirals and mAbs were well-tolerated in our experience since no serious adverse events or kidney and liver function safety issues were reported, in accordance with the results of previously published studies [5].

The totality of enrolled SOTRs obtained an antigenic negative NPS result with a median time from the beginning of treatment to NPS negativity of 13 days.

Since the study did not include a standardized protocol for assessing the time to NPS negativity and each enrolled subject performed follow-up swabs at different time points, these data are interesting but not suitable for gaining insights on the duration of SARS-CoV-2 viral shedding in SOTRs who receive an early treatment for COVID-19. Instead, the study included an accurate and defined protocol for the analysis of VL reduction in NPS from baseline through day 7 of follow-up, representing the first research which assess this virologic aspect in the group of SOTRs. These data are more intriguing if we consider that NPS viral load decrease may be considered a surrogate marker of clinical response to early COVID-19 treatment when the recognized clinical outcomes (hospitalization, ICU admission and mortality) have a low rate of incidence [13].

Surprisingly, viral load decay was similar in SOTRs treated with antiviral agents compared to those treated with mAbs. This result may be due to the limited use of nirmatrelvir/ritonavir (only one case) in the enrolled SOTRs, due to significant drug interactions with commonly used immune-suppressive medications, and support the results of a previously published study which demonstrated that nirmatrelvir/ritonavir shows the greatest antiviral activity against BA.1 and BA.2 Omicron variants [13].

The analysis of viral load kinetics revealed important factors affecting the viral load decay. Among them, anti-S positivity at baseline was associated with an increased viral load decay. If we consider that anti-S positivity is a marker of response to COVID-19 vaccination, the data confirm the pivotal role of the immunological response induced by vaccination in clearing the infection and preventing the progression to severe COVID-19 disease, even in the setting of early treatment [33]. Interestingly, SOTRs with baseline anti-S positivity experienced a significantly higher viral load decrease compared to subject with negative anti-S serology when treated with mAbs but not with antivirals. Although the study did not evaluate the immune-response of enrolled patients after COVID-19 early treatment administration, we may speculate that mAbs have additional effects beyond neutralizing the virus, enhancing the overall protective immune response in vaccinated patients, as supposed by Bang and colleagues [34]. The exact nature of this immune-stimulatory effect is not completely clear, but it likely involves antibody–antigen immune complexes that stimulate cellular immunity, possibly through Fc- effector functions [4,34].

The present study has some limitations. The study is observational and retrospective, and it therefore has limitations related to bias and confounding factors. Specifically, the allocation of treatments was based on clinical decisions rather than randomization, potentially affecting the comparability of the two treatment groups. This is a single-center study with a relatively limited sample size, which ensures accurate patient assessment but may not reflect wider practices. The study is lacking a control group who received no treatment since all symptomatic SOTRs with COVID-19 are currently offered early treatment; therefore, the findings should be interpreted with caution. The mAbs included in the study have a reduced neutralizing activity against certain Omicron sublineages [35]; therefore, the U.S. Food and Drug Administration Agency [36] and the “Agenzia Italiana del Farmaco” [37] have suspended the authorization of those mAbs for the treatment of mild–moderate COVID-19, although the in vivo efficacy is actually a debated issue [38].

## 5. Conclusions

We provide evidence of real-world effectiveness of vaccines and early therapy in SOTRs infected with SARS-CoV-2 during the Omicron wave in reducing morbidity, hospitalization and mortality. The analysis of factors affecting early SARS-CoV-2 viral load change dynamics underlines the pivotal role of the achievement of humoral response to COVID-19 vaccination in enhancing viral load decay in SOTRs. Despite the fact that the mAbs included in the study are no longer a current therapeutic option for the early treatment of COVID-19 due to the reduced neutralization activity related to the rapid and continuous emergence of new SARS-CoV-2 variants, mAbs remain a potential strategy for treating COVID-19 and other viral infections in SOTRs. The present study, by adding data on the efficacy and safety of such therapies, may be useful to support further research and development in this promising field.

## Figures and Tables

**Table 1:** Baseline characteristics of SOTRs enrolled in the study.

	N = 80 ^1^
Age, years	57.0 (48.0, 65.5)
Sex, male	48 (60.0%)
BMI, Kg/m^2^	24.5 (21.8, 27.3)
COVID-19 mRNA vaccine	
2 doses	7 (8.7%)
3 doses	50 (62.5%)
4 doses	23 (28.8%)
Days since symptoms onset	3.0 (2.0, 4.0)
eGFR, mL/min/1.73 m^2^	52.0 (38.6, 71.1)
ALT, U/L	19.0 (12.0, 35.0)
AST, U/L	26.0 (20.0, 34.0)
**Comorbidities**	
Number of comorbidities	1.0 (0.0, 2.0)
Autoimmune disease	2 (2.5%)
Cardiovascular disesase	10 (12.5%)
Cerebrovascular disease	2 (2.5%)
Diabetes	12 (15.0%)
Ematological disease	1 (1.3%)
HIV	0 (0.0%)
Chronic kidney disease	17 (21.3%)
Oncological disease	4 (5.0%)
Neurological disease	1 (1.3%)
Respiratory disease	2 (2.5%)
**Transplant**	
Kidney	50 (62.5%)
Kidney/liver	2 (2.5%)
Liver	20 (25.0%)
Heart	7 (8.8%)
Heart/kidney	1 (1.3%)
Years since transplant	5.0 (3.0, 10.5)
**Immunosuppressive drugs**	
Everolimus	10 (12.5%)
Tacrolimus	59 (73.8%)
Mycophenolate mofetil	46 (57.5%)
Cyclosporine	7 (8.8%)
Corticosteroids	45 (56.3%)

^1^ Median (Q1, Q3); n (%).

**Table 2:** Type of treatment.

Early Treatment	N = 80 ^1^
Sotrovimab	52 (65.0%)
Tixagevimab + Cilgavimib	3 (3.8%)
Molnupiravir	13 (16.3%)
Nirmaltrelvir + Ritonavir	1 (1.3%)
Remdesivir	4 (5.0%)
Casirivimab + Imdevimab	2 (2.5%)
Bamlanivimab + Etesevimab	5 (6.3%)

^1^ n (%).

**Table 3:** Univariable and multivariable linear regression analysis on factors associated with a change in SARS-CoV-2 viral load (expressed as log2 Ct values) between day 1 and day 7.

	Univariable Regression			Multivariable Regression		
Characteristic	Beta	95% CI	*p*-Value	Beta	95% CI	*p*-Value
Age, years	−0.004	−0.009, 0.002	0.2	0.000	−0.006, 0.006	>0.9
Sex						
M	—	—		—	—	
F	−0.072	−0.220, 0.076	0.3	−0.008	−0.163, 0.146	>0.9
Type of transplant						
Kidney	—	—				
Kidney/Liver	−0.108	−0.579, 0.362	0.6			
Liver	−0.118	−0.291, 0.055	0.2			
Heart	−0.071	−0.334, 0.193	0.6			
Heart/Kidney	0.274	−0.385, 0.934	0.4			
Type of early treatment						
Antivirals	—	—		—	—	
Monoclonals	−0.024	−0.198, 0.151	0.8	−0.044	−0.223, 0.136	0.6
MMF						
No	—	—				
Yes	−0.046	−0.193, 0.101	0.5			
Baseline anti-S positive						
No	—	—		—	—	
Yes	0.196	0.045, 0.347	**0.012**	0.191	0.031, 0.350	**0.020**
Baseline anti-N positive						
No	—	—				
Yes	0.002	−0.335, 0.340	>0.9			
ALT/GPT, U/L	−0.001	−0.003, 0.000	0.12			
Total bilirubine, mg/dL	−0.002	−0.016, 0.012	0.8			
eGFR, ml/min/1.73 m^2^	0.003	0.000, 0.006	0.062			
PCR	−0.021	−0.060, 0.017	0.3			
Lymphopenia						
No	—	—				
Yes	−0.058	−0.205, 0.089	0.4			
BMI, Kg/m^2^	−0.017	−0.038, 0.005	0.13			
Number of comorbidities	−0.055	−0.106, −0.004	**0.035**	−0.058	−0.110, −0.005	**0.032**
Number of immunosuppressive drugs	0.039	−0.045, 0.122	0.4			
Years since transplant	−0.006	−0.018, 0.007	0.4			
Days since symptoms onset	0.048	0.003, 0.093	**0.035**	0.050	0.006, 0.095	**0.027**
Days since last vaccination	0.000	−0.001, 0.001	0.7			

Abbreviation: CI = Confidence Interval. Bold values indicate statistical significance (*p* < 0.05).

## Data Availability

The data presented in this study are available on request from the corresponding author.

## Supplementary Materials

The following supporting information can be downloaded at: https://www.mdpi.com/article/10.3390/microorganisms13081872/s1, Table S1: Univariable and multivariable analysis of covariance (ANCOVA) on factors associated with viral load at day 7 after adjusting for viral load at Day 1.

## Abbreviations

The following abbreviations are used in this manuscript:

SOTRs	Solid organ transplant recipients
ICU	Intensive care unit
NPS	Naso-pharingeal swabs
Ct	Cycle threshold
mAbs	Monoclonal antibodies
CMIA	Chemiluminescence microparticle assay
N	Nucleoprotein
S	Spike
e-GFR	Estimated glomerular filtration rate
AST	Aspartate aminotransferase
ALT	Alanina aminotransferase
