# Supplementary material for: A Single-Center Retrospective Study on Early Treatment for COVID-19 in Solid Organ Transplant Recipients During the Omicron Era: Outcomes and SARS-CoV-2 Viral Kinetics
Source: Microorganisms. 2025 Aug 11;13(8):1872. doi: 10.3390/microorganisms13081872 (PMC12388748; doi:10.3390/microorganisms13081872)
Supplement: Supplementary file 1 [file microorganisms-13-01872-s001.zip › microorganisms-3760829_supplementary/Supplementary_material.pdf]

Table S1: Univariable and multivariable analysis of covariance (ANCOVA) on factors associated with viral load at day 7 after adjusting for viral load at Day 1.

	Univariable				Multivariable		
**Characteristic**	**N**	**Beta**	**95% CI**	**p-value**	**Beta**	**95% CI**	**p-value**
Age, years	80	0.00	-0.01, 0.00	0.13	0.000	-0.006, 0.005	>0.9
Sex	80						
M		—	—		—	—	
F		0.07	-0.08, 0.21	0.4	-0.028	-0.176, 0.120	0.7
Type of transplant	80						
Kidney		—	—				
Kidney/Liver		-0.12	-0.59, 0.35	0.6			
Liver		-0.11	-0.29, 0.06	0.2			
Heart		-0.06	-0.33, 0.20	0.6			
Heart/Kidney		0.22	-0.45, 0.89	0.5			
Type of early treatment	80						
Antivirals		—	—		—	—	
Monoclonals		-0.01	-0.18, 0.17	>0.9	-0.032	-0.202, 0.138	0.7
Mycophenolate mofetil	80						
No		—	—				
Yes		-0.07	-0.22, 0.08	0.3			
Anti-S positive at baseline	80						
No		—	—		—	—	
Yes		0.20	0.05, 0.35	**0.009**	0.207	0.056, 0.358	**0.008**
Anti-N positive at baseline	76						
No		—	—				
Yes		0.06	-0.28, 0.40	0.7			
ALT, U/L	78	0.00	0.00, 0.00	0.2			
Total bilirubine, mg/dL	79	0.00	-0.01, 0.01	>0.9			
eGFR, ml/min/1.73m^2	79	0.00	0.00, 0.01	**0.027**			
PCR	79	-0.02	-0.06, 0.02	0.3			
Lymphopenia	79						
No		—	—				
Yes		-0.08	-0.23, 0.07	0.3			
BMI, Kg/m^2	69	-0.02	-0.04, 0.00	0.087			
Number of comorbidities	80	-0.05	-0.11, 0.00	**0.039**	-0.057	-0.107, -0.007	**0.025**
Number of immunosuppressive drugs	80	0.03	-0.06, 0.11	0.6			
Years since transplant	56	0.00	-0.01, 0.01	0.8			
Days since symptoms onset	76	0.08	0.03, 0.13	**0.001**	0.086	0.038, 0.134	**<0.001**
Days since last vaccination	76	0.00	0.00, 0.00	0.8			
Day 1 viral load					0.605	0.345, 0.864	**<0.001**

Abbreviation: CI = Confidence Interval. Bold values indicate statistical significance (p<0.05)
